# Congenital Diaphragmatic Eventration in the Neonatal Period: Systematic Review of the Literature and Report of a Rare Case Presenting with Gastrointestinal Disorders

**DOI:** 10.3390/pediatric15030041

**Published:** 2023-07-26

**Authors:** Aikaterini Konstantinidi, Paraskevi Liakou, Paschalia Kopanou Taliaka, Maria Lampridou, Nicoletta Kalatzi, Ierotheos Loukas, Evangelia-Filothei Tavoulari, Konstantinos Mitropoulos, Konstantinos Koulopoulos, Rozeta Sokou

**Affiliations:** 1 Neonatal Intensive Care Unit, Nikea General Hospital “Agios Panteleimon”, 18454 Piraeus, Greece; kmaronia@gmail.com (A.K.); viviliak@gmail.com (P.L.); paschaliakt@gmail.com (P.K.T.); marialampridou1@hotmail.com (M.L.); tavoularieva@yahoo.gr (E.-F.T.); konstantinosmitropoulos@windowslive.com (K.M.); 2 Pediatric Surgery Department, Nikea General Hospital “Agios Panteleimon”, 18454 Piraeus, Greece; dot.nik@hotmail.com (N.K.); surgakis@yahoo.gr (I.L.); thkoul@yahoo.gr (K.K.)

**Keywords:** congenital diaphragmatic eventration, neonates, diaphragmatic abnormality, malformations, infants

## Abstract

Background: The term congenital diaphragmatic eventration (CDE) refers to an anatomical abnormality of the diaphragm. It is a very rare condition; however, early and prompt diagnosis is of very great importance due to possible life-threatening complications. Most severely affected patients are neonates, usually presented with respiratory distress symptoms. The aim of this study was to systematically review the existing literature and to consolidate data on CDE in neonates as well as to report a case of a neonate with congenital diaphragmatic eventration of the left hemidiaphragm and clinical signs and symptoms of the gastrointestinal tract. Methods: An electronic search of the PubMed and Scopus databases was performed regarding studies evaluating the clinical presentation, diagnosis methods, treatments, and outcomes of CDE in the neonatal population. Results: Data from 93 studies were integrated into our review, reporting 204 CDE cases, and according to them, the male/female ratio was 1/1 with a predominance of right-sided eventration. The diagnosis was primarily established by chest X-ray; surgical intervention was the most frequent treatment. The recurrence rate was 8.3% (9/109 cases). Conclusions: Early and accurate diagnosis of CDE and repair of the diaphragm can prevent complications, reduce morbidity, and improve the quality of patient’s life.

## 1. Introduction

Diaphragmatic eventration is the elevation of an intact diaphragm due to the replacement of the diaphragmatic muscle by fibroelastic tissue, either partially or completely, accounting for 5% of all diaphragmatic malformations [1]. It may be congenital or acquired. Acquired cases are more common and result from phrenic nerve injury and muscle atrophy caused by blunt or penetrating trauma, birth trauma, infection, or operative nerve injury during cervical spine surgery or thoracic surgery. Congenital diaphragmatic eventration (CDE) is a rare condition and usually unilateral, with a prevalence of 1 in 10,000 live births and male sex predominance. CDE may be isolated or co-existing with chromosomal abnormalities (especially trisomies) and congenital syndromes such as Kabuki makeup syndrome, Beckwith–Wiedemann syndrome, Poland syndrome, and Jarcho–Levin syndrome [2].

CDE could also present secondarily to congenital viral infections such as rubella and cytomegalovirus infection that cause destruction of the muscular tissue and replacement with membranous diaphragm [3,4]. Eventration can also be associated with other anomalies such as cerebral agenesis, congenital heart disease, tracheomalacia, cleft palate, malrotation, Meckel’s diverticulum, renal ectopia, and Werdnig–Hoffmann disease [5,6].

The left hemidiaphragm is more commonly affected. According to Cutler and Cooper [7], this predominance could be attributed to embryologic pathogenicity: (1) the right leaf of the diaphragm is protected by the liver, and it is stronger because of vascular concentration at this site, and (2) the left hemidiaphragm is weakened due to the atrophy of the common cardinal vein at the left side.

Most patients are asymptomatic at birth and through adult life, and diagnosis is made by a random chest X-ray; however, some of them present with significant respiratory distress or gastrointestinal symptoms. The most severely affected patients present as neonates, with signs of respiratory compromise such as mild or severe respiratory distress requiring mechanical ventilation, apnea, or a weak cry. It is of note that in some cases, the predominant signs refer to the gastrointestinal tract, including tiring during feedings and vomiting [8,9,10]. Elevated intra-abdominal organs compress the lower lobe of the lung, resulting in dyspnea, cyanosis, acute respiratory distress, vascular dysfunction, and cardiac symptoms. Wu et al. [11] reported that CDE affects the respiratory tract in the following ways: insufficient ventilation and direct compression of the ipsilateral lung; pneumonia related to chronic atelectasis; pendelluft attributed to different ventilation of the compromised and the unaffected lung; paradoxical movement of the diaphragm.

The antenatal diagnosis of CDE is very difficult as, in most cases, CDE may be confused with congenital diaphragmatic hernia (CDH). Sallout et al. [12] proposed some features that could help in differentiating these two entities: “non-deviation of the fetal heart, the presence of a thin membranous layer representing the abnormal diaphragm, changing location of the stomach on different examinations, movement of the bowel downward with fetal breathing movements, lung-to-head ratio indicative of mild disease, and presence of the ipsilateral kidney in the thorax”.

Treatment depends on the severity of the symptoms, with many surgical techniques used for the correction of the defect [13].

This study aimed at systematically reviewing the existing literature and consolidating data on CDE in the neonatal population, as well as reporting a case of a neonate with congenital diaphragmatic eventration of the left hemidiaphragm and clinical signs and symptoms of the gastrointestinal tract.

## 2. Review of Literature

### 2.1. Search Protocol/Databases

A systematic review of the literature was conducted. The keyword combination: “diaphragmatic eventration”, “congenital diaphragmatic eventration”, “eventration of diaphragm”, “neonate*”, “ newborn*”, and “fetal” with Boolean logical operators (AND, OR) was used to search PubMed and Scopus databases until May 2023 following the guidelines of the Preferred Reporting of Systematic Reviews and Meta-Analyses [14].

Furthermore, a manual electronic search and review of the references of extracted studies was performed to minimize the risk of missing studies. References of published relevant reviews were also evaluated.

### 2.2. Selection Criteria

The review included all studies and scientific articles reporting data related to CDE diagnosis during the neonatal period. We focused on studies reporting cases of neonates with CDE and especially those that describe clinical features, diagnosis methods, comorbidities, treatments, and outcomes. Studies enrolling adults and children were included if they provided relative information regarding the neonatal population separately.

Narrative reviews, systematic reviews, meta-analyses, editorials, conference papers, and studies that did not report data on neonates, as well as those published in languages other than English, were excluded. One author (R.S.) conducted the literature search and uploaded all retrieved abstracts onto the software. Three authors (P.L., K.M., E.T.) independently screened abstracts to identify eligible studies and reviewed the selected articles in full text, while another author (A.K.) resolved any disagreement.

## 3. Results

**Figure 1:**
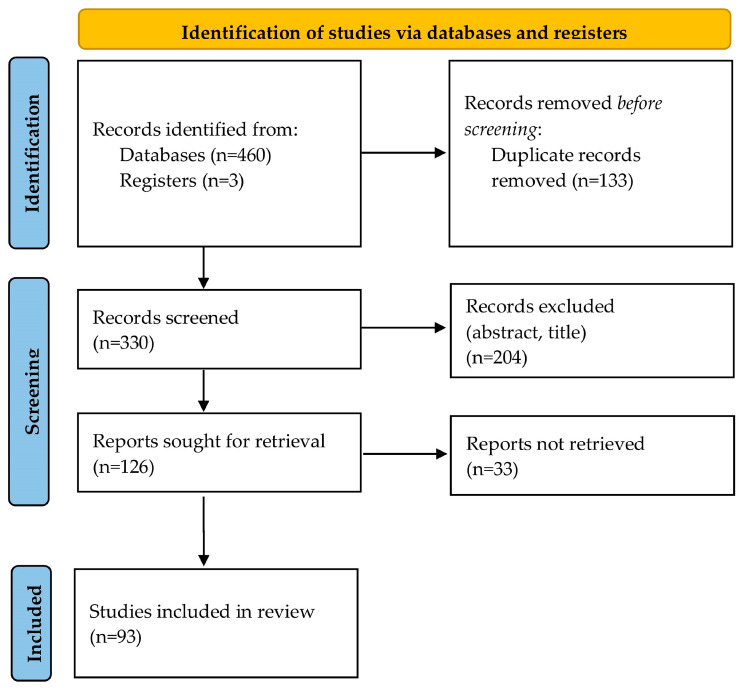
Flowchart of study selection process.

A total of 463 studies were extracted from the initial search of the literature databases; 133 of them were duplicates and were removed. After reviewing the title and the abstract, 204 studies were excluded. After thoroughly reading and evaluating the full text of the remaining 126 articles, 93 studies met the inclusion criteria and were included in the review (reference list of included studies is presented in the supplementary material). A study flowchart is presented in Figure 1. From a total of 93 eligible articles, 204 cases of neonates with CDE were reported. A summary including the characteristics of the reported cases is presented in Table S1.

Among 204 cases reported, data regarding gestational age were provided in 138 cases; 102 (73.9%) were term neonates and 36 (26.1%) were preterm neonates. Based on available data (177 cases), the male/female ratio was 1/1. Left-sided eventration was reported in 54 (28.9%) cases, right-sided in 92 (49.2%) cases, and bilateral in 39 (20.9%) cases, while in 2 (1%) cases, the eventration was central. In 54% of cases, the symptoms appeared during the first 3 days of life. Regarding the clinical presentation, 188 neonates presented respiratory distress, 6 neonates had symptoms from the gastrointestinal tract, 11 neonates were asymptomatic, and in 22 cases data were not provided. In 133 out of 163 cases with available data, comorbidities were reported with a predominance of congenital heart diseases and congenital malformations. Only in 24 cases was the diagnosis of CDE suspected prenatally; chest X-ray and CT were the most used diagnosis methods. Regarding the therapeutic choices, surgical intervention was the most frequent treatment; only in 25 cases the patients were treated conservatively. In 142 (72%) cases out of 197 (in 7 cases data were not reported) the outcome was good, while the mortality rate was 28% (55 cases). The recurrence rate, according to retrieved data, was 8.3% (9/109 cases).

## 4. Case Presentation

A male neonate with gestational age of 40^+1^-weeks was born to a 28-year-old secondi gravida mother via vaginal delivery, with a birth weight of 3.420 g. The antenatal period was uneventful, as was confirmed by the ultrasound screening tests and another workup. The newborn cried immediately after birth, and there was no need for special resuscitation, so he was given to his mother to breastfeed. The postpartum period during the hospital stay was uneventful, except for a brachial plexus paresis due to a right clavicle fracture as a result of shoulder dystocia. The neonate was discharged from the hospital at 4 days of life. The following days at home, the neonate presented episodes of intermitted vomiting after feeding whenever the milk feed volumes exceeded 35 mL. The abdominal ultrasound suggested by the pediatrician showed no findings for intestinal torsion or a typical picture of pyloric stenosis, although incomplete gastric torsion could not be ruled out.

**Figure 2:**
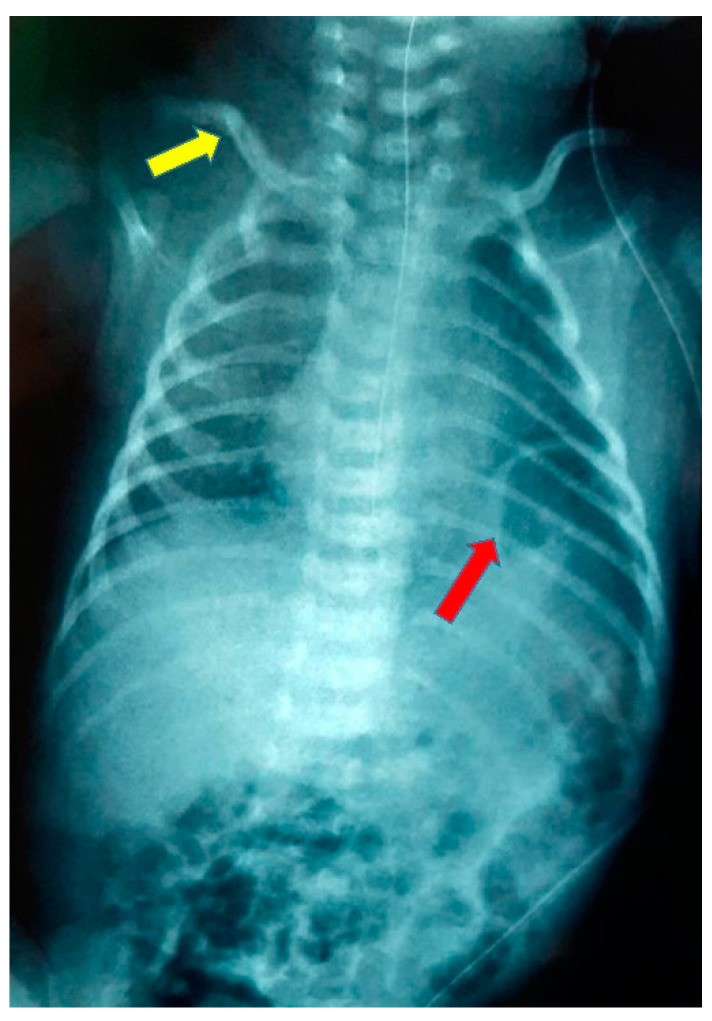
Chest radiograph at admission showing elevation of the left hemidiaphragm (red arrow) and right clavicle fracture (yellow arrow).

At 8 days of life, the mother was referred to our neonatal intensive care unit (NICU) for further investigation as vomiting persisted. Clinical examination revealed a fixed right clavicle fracture and brachial plexus paresis, while the vital signs were normal, and there were no signs of respiratory difficulty. The laboratory assessment showed: hemoglobin 14.4 gm%, hematocrit 40.3%, total leucocyte count 12,890 k/μL, polymorphs 21.3%, monocytes 0.5%, lymphocytes 54.8%, platelets 449.000 k/μL, sodium/potassium 139/5 mmol/l, calcium 10.2 mg/dl, total bilirubin 6.3 mg/dl, C-reactive protein negative, and arterial blood gases were normal. Both chest and abdominal X-rays showed elevation of the left hemidiaphragm with the possible existence of a hernia sac and the presence of intestinal helices in the left hemithorax (Figure 2).

Although the infant did not present with respiratory distress at that time, diaphragmatic palsy, congenital diaphragmatic hernia, or diaphragmatic eventration were considered possible diagnoses. The pediatric surgery team conducted a passage with a radiographic contrast agent that revealed the presence of stomach and left colic bend in the left hemithorax with an elevation of the left hemidiaphragm; surgery was decided. Cranial and abdominal ultrasounds performed before surgery were without pathological findings. Enteral feeding was discontinued, and intravenous parenteral fluids were administered along with antibiotic therapy with cefoxitin, gentamicin, and metronidazole as a preparation for the surgical intervention.

**Figure 3:**
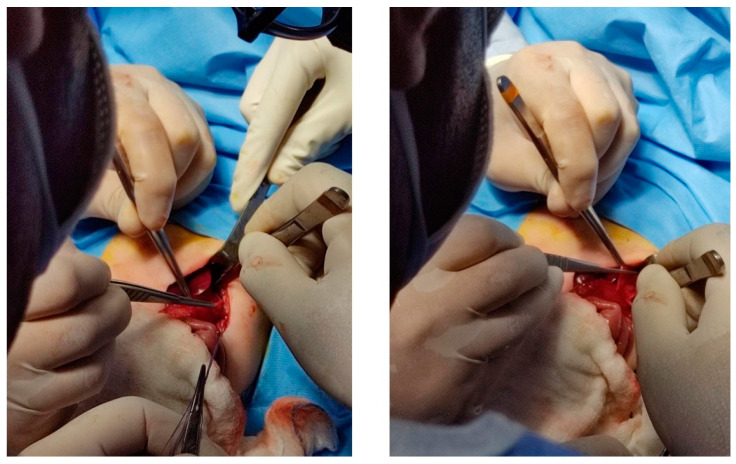
Intraoperative procedure.

A transabdominal repair was performed. During surgery, a left supra-umbilical transverse incision was made, and a high-grade atrophy of the central portion of the diaphragm with significant elevation showed up. The left lobe of the lung appeared to be compressed. The sac contained the spleen, stomach, left colic bend, left lobe of the liver, and a part of the right lobe of the liver. After reducing the herniated organs into the abdominal cavity, diaphragmatic plication was performed (Figure 3).

After surgery, the neonate was transferred to the NICU. The postoperative period was uncomplicated, and the neonate was extubated on the 3rd postoperative day without the need for oxygen supplementation. Continuous enteral feeding started on the 4th postoperative day with a gradual increase in the amount of milk given. Cranial, kidney, and abdominal ultrasounds were normal. Full enteral feeding without any problem was achieved on the 12th postoperative day, and the infant was discharged from the NICU on the 23rd day of life, weighing 3640 g. Instructions were given to the parents to perform chest and abdominal X-rays at the age of 3, 6, and 12 months of life, abdominal ultrasound after 1 month and at the age of 3 months, as well as cardiological evaluation.

**Figure 4:**
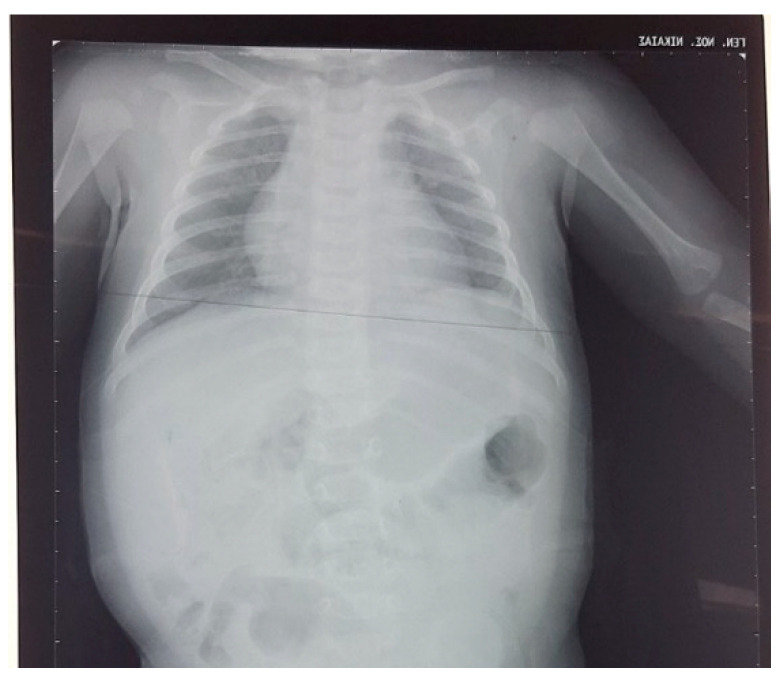
Chest radiograph at the age of 3 months.

The abdominal ultrasound performed 1 month later was without pathological findings. The chest/abdominal X-rays at the age of 3 and 6 months were normal, and there were no signs indicating a rebound of the eventration of the diaphragm (Figure 4). The right brachial plexus paresis was resolved at the age of 6 months. The infant had adequate growth, and no feeding problems were reported from the parents.

## 5. Discussion

CDE as an anomaly of the diaphragm was initially described by Jean Louis Petit in 1774 as “a special form of thoracic hernia” during a postmortem examination, but the term diaphragmatic eventration appeared in the literature for the first time by Beclard and Cruveilhier in 1829 and became generally accepted instead of definitions such as diaphragmatic relaxation, phrenic insufficiency, and hypertrophied diaphragm [11,15,16,17]. In 1947, Bisgard reported the difference between CDH and CDE, and according to this report, CDE is “an abnormally high, or elevated position of one leaf of the diaphragm as a result of paralysis, aplasia, or atrophy of varying degree of the muscle fibers. Its unbroken continuity differentiates it from diaphragmatic hernia” [18]. Chin and Lynn, in 1956, reported an incidence of 1 in 1400 patients in an X-ray survey [15,19]. Until the 20th century, eventration was a rare diagnosis in infants; data regarding this entity came mainly from necropsies (BayneJones, 1916; Reed and Borden, 1935) [20,21]. Since the 21st century, the evolution of diagnostic methods and treatment management has led to an increased number of diagnosed and reported cases of CDE in children with good outcomes [11].

Embryologically, the diaphragm is derived from muscular components and three more elements: the septum transversum, the pleuroperitoneal membranes, and the mesentery of the esophagus. Eventration of the diaphragm can be congenital or acquired [11,15]. CDE is present at birth as the result of inadequate muscularization of the fused pleuropericardial membrane that builds the diaphragm [22].

The inadequate muscularization of part of or the entire hemidiaphragm is presented as diaphragmatic eventration, and the most acceptable theory for the etiology of this condition is the altered myoblast migration to the septum transversum and the pleuroperitoneal membrane [23]. The muscular segment of the diaphragm in the microscope seems to be sparsely distributed and nonfunctional but not atrophic [1,6,24]. The characteristic element in the histological examination of the diaphragm is the lack of muscular fibers and diffuse fibroelastic changes [25]. All these disturbances result in a thin hemidiaphragm, elevated like a smooth membrane into the involved hemithorax.

The thinned, weakened hemidiaphragm is insufficient to hold the abdominal organs; the diaphragm goes up and drags them into the thoracic cavity. Although in the general population, left-sided eventration appears to be most frequent (9:1) with a two-fold predominance of males [16], according to our systematic review, in neonates, the right-sided CDE seems to predominate with a male/female rate of 1/1. The predominance of the right-sided CDE in infants and children reported by Robinson et al. [26] was hypothesized to be attributed to the left-side reinforcement by the heart and pericardium, although the precise pathophysiological mechanism is unclear. It is well known that diaphragmatic defects are diffused or partial, with the latter mostly affecting the right hemidiaphragm [11,27,28]; in the studies included in our systematic review, there was no report on whether the defect was diffused or partial, so it is uncertain if the predominance of the right-sided eventration found is related to a partial defect of the diaphragm. Eventration of the diaphragm may occur as a secondary diaphragmatic paralysis (absence or injury of the phrenic nerve) due to birth trauma, congenital infection (polio, cytomegalovirus, and rubella), tumor infiltration of the phrenic nerve, and operative injury during cervical spine surgery. In infants, the most common cause of acquired eventration is birth trauma, attributed in most cases [15,27,29,30] to difficult vaginal delivery with shoulder dystocia that may lead to a stretch injury of the C3–C5 roots and affect the origin of the phrenic nerve and brachial plexus (Erb–Duchenne or Klumpke’s palsy). Clavicle fractures may be present at the same hemithorax with the eventration of the hemidiaphragm. It is noteworthy that in our case, the newborn had a clavicle fracture that, however, was present on the opposite side of the eventrated hemidiaphragm. It is difficult to estimate with accuracy the incidence of acquired diaphragmatic eventration because many cases are asymptomatic and not reported in the literature.

Clinical presentation of eventration of the diaphragm varies in severity, and the patients often appear to be asymptomatic. In most of the reported cases in the literature, clinical presentation occurred mainly with respiratory distress (tachypnoea, dyspnea, decreased breath sounds, paradoxical movement of the chest), cyanosis, cardiac symptoms, and gastrointestinal symptoms such as vomiting, anorexia, nausea, bowel sounds into the thoracic cavity, scaphoid abdomen, and abdominal pain [6,11,13,15]. During the neonatal period, respiratory symptoms dominate, and this finding is in line with our review results. In our case, the infant appeared to be asymptomatic at birth, but in the following days, he presented gastrointestinal symptoms such as quantity-related vomiting.

CDE must be differentiated from other situations like CDH with sac, phrenic nerve injury, myotonic dystrophy, and post-GBS infection [11]. Maternal, obstetric, and perinatal history could help to exclude acquired eventrations. It is also of great importance to distinguish antenatally, if possible, CDE from CDH, as the latter is associated with higher morbidity and mortality [31]. Although the diaphragm is intact in CDE, unlike CDH, the antenatal differentiation between these two entities by ultrasonographic examination is challenged because of similar imaging findings [12,24]. Instead, magnetic resonance could help with early in utero diagnosis of CDH [31]. Sallout et al. [12] propose the following features to prenatally differentiate the two pathologies performing serial ultrasonographic examinations: “non-deviation of the fetal heart, the presence of a thin membranous layer representing the abnormal diaphragm, changing location of the stomach on different examinations, movement of the bowel downward with fetal breathing movements, lung-to-head ratio indicative of mild disease, and presence of the ipsilateral kidney in the thorax”.

Usually, the diagnosis of CDE is not suspected prenatally, and even after birth, it may be delayed due to the absence of symptoms [11,32]. Our review results showed that CDE was suspected prenatally only in 24 cases, but there was difficulty in differentiating from CDH, and the final diagnosis of CDE was confirmed postnatally.

Postnatally, chest radiographs, frontal and lateral, can set the diagnosis of CDE by displaying characteristic elevation of the affected hemidiaphragm for at least two intercostal spaces higher than the unaffected side, and they may highlight bowel helices into the thoracic cavity [9]. Nevertheless, the differential diagnosis between CDH and CDE remains difficult since radiographic findings, such as mediastinal shift, the appearance of stomach or bowel in the thorax with no bowel gas in the abdomen, are the same for both entities in most cases. Ultrasound can be used as a diagnostic method by demonstrating minimal or paradoxical movement of the diaphragm [11,15,33]. Finally, in most cases, the diagnosis is confirmed intraoperatively, as it happened in our case; the diaphragm was found to be not well developed and atrophic, although intact.

Treatment of CDE is mainly surgical, especially when severe respiratory distress with the need for mechanical ventilation or failure-to-thrive is present [15,29]. The most common surgical intervention is diaphragmatic plication, performed with various techniques and approaches (open transthoracic, thoracoscopic, open transabdominal, and laparoscopic approaches). The purpose of the plication treatment is to minimize the abundant diaphragmatic surface and lower the hemidiaphragm [11,34]. The choice between thoracoscopy versus thoracotomy approach depends on the size of the patient, the clinical presentation, and the surgeon’s expertise. Furthermore, there is a less invasive procedure: video-assisted thoracic surgery (VATS). The choice of surgical approach technique depends on the skills and experience of the staff at each center.

Complications of the surgical interventions include wound infection, adhesive intestinal obstruction, partial wound dehiscence, recurrence and reoperation, pneumothorax, and empyema [10].

Early and accurate diagnosis of CDE and repair of the diaphragm can prevent complications from gastrointestinal and respiratory tracts and improve the quality of a patient’s life [11,35].

The patients who went through diaphragm plication surgery should be monitored for possible complications or recurrence of the eventration [13]. Based on our review data, the recurrence rate was 8.3%. The long-term outcome of these patients is usually good [11,15], a finding confirmed by our review results. The mortality rate was found to be 28% in our literature review, but it probably could be attributed to coexisting morbidities. Whenever the clinical presentation is delayed, the prognosis is usually better, and this fact may be attributed to absent or mild lung hypoplasia [6,36].

The management and the prognosis of diaphragmatic eventration depend on the severity of clinical status and the underlying etiology; acquired elevation of the diaphragm due to birth trauma has the potential to resolve automatically [13].

## 6. Conclusions

Diagnosis of CDE is challenging, and it can be delayed due to the variability of its clinical presentation; however, neonatologists should suspect CDE in every infant with respiratory distress and an X-ray suggestive of lower lobe opacity. The surgery team should be in collaboration with neonatologists to manage the situation and ensure the best outcome for the patient. Follow-up is essential for these patients as diaphragmatic eventration has the tendency to relapse.

## Data Availability

Data are contained within the article.

## Supplementary Materials

The following supporting information can be downloaded at: https://www.mdpi.com/article/10.3390/pediatric15030041/s1, Table S1: characteristics of the reported cases.
