# Supplementary material for: Congenital Diaphragmatic Eventration in the Neonatal Period: Systematic Review of the Literature and Report of a Rare Case Presenting with Gastrointestinal Disorders
Source: Pediatr Rep. 2023 Jul 26;15(3):442–51. doi: 10.3390/pediatric15030041 (PMC10443257; doi:10.3390/pediatric15030041)
Supplement: Supplementary file 1 [file pediatrrep-15-00041-s001.zip › pediatrrep-2403253-supplementary.pdf]

Table S1. Characteristics of the reported cases.

Author, Year	Patients	Gestational age	Gender	Symptoms onset time (days)	Clinical signs	Side	Comorbidity	Diagnosis method	Treatment	Patient outcomes	Recurrence
Singh, 2020 [1]	9	N/A	N/A	6 (3–26)	N/A	Mostly left	N/A	N/A	Surgical	Good	N/A
Heiwegen, 2020 [2]	17	Median 38	Male 8/17	N/A	Dysmorphic characteristics	Right/left 10/5	N/A	Prenatal ultrasound	Surgical	One death	Three cases
Hoshino,2020 [3]	1	36	Male	1	Respiratory failure	Left		Fluoroscopic examination	Surgical	Good	No
Iskender, 2012 [4]	1	37	Male	1	Peripheral cyanosis	Right	Non-immune hydrops fetalis	MRI	Conservative	Good	N/A
Kadam, 2017 [5]	1	40	Male	2	Respiratory distress	Left	Gastric volvulus	Chest skiagram	Surgical	Good	No
Kizilcan, 1993 [6]	1	N/A	Female	N/A	Tachypnea, dyspnea, cyanosis	Right	N/A	Fluoroscopy	Surgical	Good	No
Lai, 2010 [7]	1	Term	Female	1	Bilious vomiting with abdominal distension	Right	Long jejunal duplication, right upper-limb amelia	Chest computed tomogram	Surgical	Good	No
Gunadi, 2020 [8]	1	Term	Male	16	Respiratory distress	Right	No	Chest X-ray	Surgical	Good	No
Kasdallah, 2017 [9]	1	Term	Male	3	Distended abdomen with generalized contracture, bilious gastric residuals	Left	No	Ultrasonography	Surgical	Good	No
Mitsiakos, 2022 [10]	1	32	Female	1	Respiratory distress	Right	Congenital cardiac anomalies and parvovirus B19 infection	U/S and chest X-ray	Surgical	Death	
Revencu, 2004 [11]	1	Term	Male	1	Respiratory distress	Right	Marfan syndrome	U/S and chest X-ray	Conservative	Death	

Sallout, 2021 [12]	1	Term	N/A	1	Asymptomatic	Left	No	U/S and chest X-ray	Surgical at 6 months	Good	No
Tsukahara, 2001 [13]	1	Term	Female	1	Respiratory distress	Left	No	Prenatal MRI	Conservative	Good	
Yang, 2002 [14]	1	Term	Female	1	Respiratory distress	Left	No	Chest X-ray	Surgical	Good	No
Zankl, 2007 [15]	2	30	Female	1	Floppy and cyanotic	Right	Non-immune hydrops fetalis	Autopsy after death	Conservative	Death	
Lewis, 2008 [16]	1	Term	N/A	19	History of poor suck, inconsolable cry, and hurried breathing	Left	Wolff–Parkinson–White (WPW) syndrome	Chest X-ray	Conservative	Good	N/A
Shukla, 2012 [17]	1	42	Male	1	Respiratory distress since birth	Left	Two gastric duplication cysts	Ultrasonography of the thorax	Surgical	Good	No
Sathanandam, 2016 [18]	1	40	Male	1	Severely hypoxic	Left	Congenital cardiac anomalies, congenital diaphragmatic hernia	Computed tomography	Surgical	Good	No
Shehata, 2000 [19]	1	Term	Male	18	Respiratory problems	Left	Congenital skeletal anomalies	Fluoroscopy	Surgical	Good	No
Steiner, 2002 [20]	1	37	Female	1	Hypoxemia	Bilateral	Persistant pulmonary hypertention, dysmorphic characteristics	Chest X-ray	Conservative	Death	
	1	38	Male	1	Respiratory distress	Right	Anophthlamia, dysmorphic characteristics,	Chest X-ray	Surgical	Death	

							cryptorchidism bilateral inguinal hernia				
Stratton, 1993 [21]	1	37	Female	1	Cyanosis	Bilateral	Tetralogy of Fallot, Fryns syndrome	U/S	Surgical	Death	
Symbas, 1976 [22]	1	Term	Female	1	Severe respiratory insufficiency	Right	No	N/A	Surgical	Good	No
	1	Term	Female	1	Severe respiratory insufficiency	Right	No	N/A	Surgical	Good	No
	1	Term	Female	21	Severe respiratory insufficiency	Right	Right Erb's palsy, aortic stenosis	N/A	Surgical	Death 5 wk postoperative due to severe aortic stenosis	
	1	Term	Male	2	Asymptomatic	Right	Multiple other anomalies	N/A	Conservative	Good	N/A
	1	Term	Female	20	Upper respiratory infection	Right	N/A	N/A	Conservative	Good	N/A
	1	Term	Female	2	Asymptomatic	Right	Turner's syndrome, coarctation of aorta	N/A	Conservative	Good	N/A
	1	Term	Male	28	Nausea and vomiting	Left	No	N/A	Surgical at 4 years	Good	
	1	Term	Male	7	Failure to thrive and dyspnea, severe respiratory insufficiency	Bilateral	VSD and PDA	N/A	Surgical	Good	No
Takahashi, 2014 [23]	1	38	Male	3	Respiratory failure	Bilateral	HLHS, omphalocele, PAGOD syndrome	Fluoroscopy and computed tomography	Surgical	Death	
Martins, 2021 [24]	1	Term	Female	1	Acute respiratory distress	Right	Scimitar syndrome	CT scan	Surgical	N/A	N/A
Vijayaraghavan, 1998 [25]	1	Term	Female	1	Severe respiratory distress	N/A	N/A	N/A	N/A	N/A	N/A

Wayne, 1973 [26]	1	Term	Female	3	Severe respiratory distress	Bilateral	CMV congenital infection	Chest X-ray	Conservative	N/A	N/A
Byun, 2014 [27]	1	Term	Female	1	Vomiting, dyspnea	N/A	N/A	N/A	Surgical	Good	N/A
	1	Term	Female	5	Vomiting, dyspnea	N/A	N/A	N/A	Surgical	Death	N/A
Snyder, 2014 [28]	5	Preterm	-	N/A	Respiratory distress	Right (4/5)	Myotonic dystrophy	Chest radiographs	Surgical	Good	No
Rattan, 2001 [29]	1	Term	Female	3	Respiratory distress	Right	Lung agenesis	Chest radiographs, adbominal ultrasound	Surgical	Death, 24 hours postoperative	
Rais-Bahrami, 1996 [30]	1	40	Female	1	Respiratory distress	Right	Segment of the 9 and 10 ribs, hypoplastic right lung	Chest radiographs, ultrasound, autopsy	Surgical	Death	
Sciorra, 1992 [31]	1	39	Male	1	Fetal growth retardation, dysmorphic features, respiratory distress	N/A	Trisomy 5 mosaicism	N/A	Surgical	Death	
Yang, 2005 [32]	1	41	Female	1	Cyanosis	Right	Right lung hypoplasia	Chest radiographs, CT, prenatal	Surgical	Good	No
Poole, 1976 [33]	1	Term	Female	1	Respiratory distress	Left	Cleft lower sternum and small ventral hernia, congenital heart disease	Chest radiographs, autopsy	Surgical	Death 24 h postoperative	
Kapoor, 2010 [34]	1	33+5	Male	1	Repsiratory distress, hypotonia	Right	Myotonic dystrophy	Chest radiographs, ultrasound	Conservative	Death at 40 days of life	
Pearl, 1972[35]	1	40	Male	1	Tachypnoia, cyanosis	Left	Extralobar sequestration with several bronchioles and pulmonary parenchyma	Chest radiographs	Surgical	Good	No
Renault, 2008 [36]	1	39	Male	1	Generalized hypotonia, respiratory distress	Bilateral	No	Chest radiographs	Surgical plication	Death at 11 months	

	1	38	Male	1	Birth asphyxia	Bilateral	No	Chest radiographs	Surgical	Bronchial and pulmonary infections	No
Moerman, 1987 [37]	1	34+5	Male	1	Hypotonia	Bilateral	X-linked centronuclear myopathy	Autopsy	N/A	Death after 30 min resuscitation	
	1	42	Female	1	Asphyxia	Bilateral	Pulmonary hypoplasia, myotonic dystrophy	Autopsy	N/A	Death	
Moccia, 1981 [38]	1	Preterm	N/A	1	Respiratory distress	Right	Type 3 truncus arteriosus, duodenal atresia	Chest radiographs, ultrasound	Surgical	Death	
	1	N/A	N/A	21	Respiratory distress	Right	N/A	Chest radiographs, ultrasound, radionuclide liver–spleen scan	N/A	Good	N/A
Moazamm, 1979 [39]	1	N/A	Female	14	Respiratory distress	Bilateral	N/A	N/A	Surgical	Good	No
Gupta, 2006 [40]	1	35	Female	1	Poor respiratory effort	Right	Pulmonary hypoplasia, mitochondrial dysfunction	Autopsy	Surgical	Death at 23 h	
	1	35	Female	1	Repsiratory distress	Bilateral	Absence of 12th rib, pulmonary hypoplasia, mitochondrial dysfunction	Chest radiographs, autopsy	N/A	Death at 26 days	
	1	35	Male	N/A	N/A	Left	Type 3 truncus arteriosus and ventricular septal defect, mitochondrial dysfunction	N/A	Surgical	Death at 55 days	
Merten, 1982 [41]	1	N/A	Female	1	Mild cyanosis, tachypnoia	Left	Trisomy 13–15	Chest radiographs, autopsy	N/A	Death at 26 days	

	1	N/A	Male	3	Respiratory distress	Bilateral	Short-arm translocation of chromosome 22	Chest radiographs	N/A	Good	No
	1	N/A	Male	1	Respiratory distress	Bilateral	Fetal alcohol syndrome	Chest radiographs, surgery	Surgical	Death at 3 months of age (massive aspiration)	
	1	N/A	Female	1	Mild respiratory distress	Right	Aural pinna, nasal septum malformation, and bilateral hypoplasia of the 5th finger	Chest radiographs, ultrasound	N/A	At 4 months of age developmental delay, mild hypertension	N/A
Meine, 1974 [42]	1	N/A	Female	N/A	Respiratory distress	Left	Multiple congenital anomalies	Chest radiographs	Surgical	Transient hypertension	Yes
Macayran, 2002[43]	1	35+2	Female	1	Respiratory distress	Right	PAGOD syndrome	Chest radiographs, ultrasound, CT	Surgical	Death at 6 months	
Kozlov, 2015 [44]	35	N/A	Male/female 20/15	N/A	Respiratory disorders	Right/left side 24/11	13 cases with cardiac malformations and genetic malformations	Chest radiographs	Surgical	Good	2 cases
Joshi, 2018 [45]	1	Term	Female	4	Breathing difficulty, subcostal retractions, reduced breath sounds left	Left	No	Chest radiographs	Surgical	Right-sided evantration postoperative, tract infections recurrent	No
Saravanan, 2015 [46]	1	N/A	Female	6	Respiratory distress	Bilateral	N/A	Chest and abdomen radiographs, ultrasound, CT	Surgical	Second plication on the right side, discharged after 5 days	No
Heo, 2017 [47]	1	36+5	Male	1	Shallow excursion, weak diaphragmatic motion	Right	Marfan, dysmorphic features, dilated aortic	Chest radiographs, ultrasound	N/A	Good	N/A

							root, hydronephrosis, hydroureter right kidney, dilated cisterna magna				
Blanluet, 2010 [48]	1	Term	Male	10	N/A	Right	Prune-belly sequence	Radiographs, ultrasound	Surgical	Good	No
Garbaccio, 1972 [49]	1	Term	Female	2	Respiratory distress, tachypnoia, cyanosis	Left	No	Radiograph	Surgical	Good	No
	1	Term	Female	1	Tachypnoea in birth, bilateral atelectasis at birth,small feeding in upright position, fatigue, poor weight gain, atrophy and paralysis of the muscles in right arm, thoracic scoliosis	Right	N/A	Radiograph, fluoroscopy	Surgical	Atelectatic lung, expansion after 4 postoperative days; 4 years normal lung function but limited function of right extremity	No
	1	41	Male	7	Respiratory distress, cyanosis which worsened with feedings, mild Erb's palsy of the left side, and a right phrenic nerve paralysis. Difficulty in delivery of head and left shoulder	Right	Erb's palsy left-sided	Radiograph, fluoroscopy	Surgical	Good	No
	1	43	Female	6	Respiratory distress, tachypnoia, cyanosis, heart sounds on the right, breath sound decreased on left, during feeding worst	Left	Phocomelia left and right, complete agenesis left phrenic nerve, agenesis of left lower lobe of the lung	Radiograph, fluoroscopy	Surgical	Good	No

Falade, 1992 [50]	1	Term	Female	10	Respiratory distress, vomiting and refusing feeds, decreased breath sound on right	Right	Abnormal liver configuration	Radiograph, autopsy, right-sided diagnostic chest tap, right closed thoracostomy tube drainage	Conservative	Death 14 days of live	
Ertan, 2010 [51]	1	N/A	Female	1	Asymptomatic	Right	Multiple congenital abnormalities	prenatal ultrasound CDH, chest radiograph, CT	Surgical	N/A	N/A
	1	Preterm	Female	1	Prenatal diagnosis	Right	Omplhalocele, skeletal anomalies	Prenatal ultrasound, chest radiographs	Surgical	Good	N/A
Elberg, 1989 [52]	1	36	Male	1	Severe respiratory distress	Bilateral	Hypoplastic lungs	X-ray	Conservative	Death 10 hours	No
	1	36	Male	1	Severe respiratory distress	Bilateral	Hypoplastic lungs	X-ray	Conservative	Death 14 days of live	
Ekkelkamp, 1983 [53]	1	37	Female	1	Birth asphyxia	Bilateral	No	Fluoroscopy	Surgical	Good	No
Rana, 2021 [54]	1	37	Male	1	No symptoms, prenatal diagnosis	Left	No	Fetal ultrasound, fetal MRI, postnatal CT	Surgical	Good	N/A
Dalvi, 1990 [55]	1	N/A	Female	1	Tachypnoea	Right	No	X-ray, CT scan	Surgical	Good	N/A
Krasemann, 2020 [56]	1	N/A	Male	1	Cyanotic	Right	Marfan syndrome, clockwise rotated heart, valvar disfuntcion, muscular hypotonia	X-ray	Surgical	Death at 10 weeks	
Comstock, 1986 [57]	1	38	N/A	1	Respiratory distress	N/A	Trisomy 18, horseshoe kidney	Fetal ultrasound at 35 w	N/A	Death at 2 days	
Wenger, 1997 [58]	1	N/A	Male	1	Apnoea, poor respiratory effort, feeding problems	Left	Chromosomal anomalies	X-ray, fluoroscopy	Conservative	Death at 2 months at home	
Donahue, 2017 [59]	1	32+3	Male	1	Respiratory distress	Left	Chromosomal anomalies	X-ray	Surgical	Respiratory insufficiency progressing to respiratory	N/A

									failure tracheostomy at 9 months	
Othersen, 1977 [60]	1	Preterm	Male	1	Respiratory distress	Right	N/A	X-ray, fluoroscopy, liver scan	Surgical	Death at 70th day	
	1	N/A	Male	9	Mild respiratory distress	Right	N/A	X-ray, intraoperative	Surgical	Good	N/A
	1	Preterm	N/A	1	Respiratory distress	Left	Patent ductus arteriosus (PDA)	X-ray	Surgical	Good	Small diaphragmatic elevation at 9.5 months
Ein, 1997 [61]	1	39	Male	1	N/A	Right	Anal agenesis with rectal and sigmoid atresia	X-ray	N/A	N/A	-
Berdon, 1968 [62]	1	Term	N/A	1	Respiratory distress	Right	Bilateral pulmonary hypoplasia	X-ray, autopsy	Surgical	Death	-
	1	Term	N/A	1	Respiratory distress	Left	Bilateral pulmonary hypoplasia	X-ray, autopsy	Surgical	Death	-
	1	Term	N/A	1	Respiratory distress	Right	Unilateral pulmonary hypoplasia	X-ray	Surgical	Death	No
Baharin, 2020 [63]	1	34	Female	1	Repsiratory distress	Right	Poland syndrome, Sprengel's deformity, hypoplasia of right chest wall	X-ray, ultrasound, CT	Surgical	Assymetry of the chest wall	No
Buxi, 1996 [64]	1	Term	Male	1	Respiratory distress	Right	Aplasia of the right lung	X-ray, CT scan	Surgical	Death	-
Iliff, 1982 [65]	1	41	Male	1	Tachypnoea	Central	Pericardial effusion	X-ray, fluoroscopy, intraoperative	Surgical	Good	N/A
Rao, 2016 [66]	1	Term	Female	2	Respiratory distress, periferic cyanosis	Bilateral	No	X-ray, CT scan	Surgical	Good	N/A

Andijani, 2019 [67]	1	38	Male	1	Respiratory distress	Left	PDAC syndrome/microphthalmic syndrome 9	X-ray, CT, fluoroscopy	Conservative	Ventilator depended tracheostomy	N/A
Baker, 2023 [68]	1	38	Female	1	Severe respiratory failure, pulmonary hypertension	Bilateral	King–Denborough syndrome (KDS), central core disease (CCD)	Fetal MRI, CT, autopsy	Conservative	Death at 5 months	-
Chitayat, 2007 [69]	1	38	Female	1	-	Left	PDAC syndrome	Fetal ultrasound 24 w, autopsy	N/A	Death shortly after delivery	-
	1	Term	Male	1	-	Right	PDAC syndrome	Fetal ultrasound 34 w	N/A	Death shorty after delivery	-
	1	31+6	Female	1	Repsiratory distress	Bilateral	Multiple congenital abnormalities	Autopsy	N/A	Death at 50 min of life	-
	1	41	Male	2	Respiratory distress	Left	Multiple congenital abnormalities	X-ray, CT	Surgical	Delayed development and truncal hypotonia	No
	1	36	Male	1	Cyanotic	Right	Multiple congenital abnormalities	X-ray, autopsy	N/A	Death at 3 days of life	-
Kosho, 2006 [70]	1	36+2	Male	1	Heart and respiratory failure	N/A	Trisomy 18, double outlet right ventricle (DORV)	N/A	N/A	Death	-
	1	31+4	Male	1	Respiratory failure	N/A	Trisomy 18, IUGR, lung hypoplasia, trancheosophageal fistula	N/A	N/A	Death at 1 day of life	
	1	38+3	Female	N/A	N/A	N/A	Trisomy 18, IUGR	N/A	N/A	Good	N/A

Robinson, 1980 [71]	1	35	Male	16	Respiratory distress	Bilateral	No	X-ray	Surgical	Postoperative X-ray had minimal changes	N/A
	1	N/A	Male	7	Mild respiratory distress	Bilateral	Hypospadias skeletal anomalies. VSD, valvular pulmonic stenosis	X-ray	Conservative	Good	No
	1	N/A	Male	1	Respiratory distress	Bilateral	PDA, aortic coartation, pulmonary hypoplasia	Autopsy	N/A	Death	
Miyano, 2015 [72]	1	34	Male	1	N/A	Right	Unilateral pulmonary agenesia, oesophageal atresia and tracheoesophageal fistula	Fetal ultrasound 22 w, X-ray	Surgical	Good	No
Arts, 2020 [73]	1	38	Female	1	Persistent pulmonary hypertension	Bilateral	CAKUT syndrome, pulmonary hypoplasia, congenital alveolar dysplastic pattern	Autopsy, fetal ultrasound 16 week		Death at 1 day of life	-
	1	35	Female	Stillbirth		Bilateral	Mutation in PBX1, small kidneys and bilateral ureteric dilatation, a bell-shaped chest, and pulmonary	Fetal ultrasound 16 w, autopsy		Stillbirth	-

							hypoplasia, sex reversal 46XY				
Wexler, 1976 [74]	1	39	Female	1	Hypoxia	Bilateral	No	Fluoroscopy, autopsy	Surgical	Good	
	1	37	Female	1	Respiratory distress	Right	No	X-ray, fluoroscopy	Conservative	Good	Persistent elevation of the right hemidiapharg m
	1	40	Female	1	Cyanosis	Bilateral	Trisomy 13–15	X-ray, autopsy	N/A	Death at 2 days of life	-
	1	34	Female	1	Asymptomatic	Bilateral	Trisomy 18, hypoplastic lungs	X-ray, autopsy	N/A	Death at 4 days of life	
	1	35	Male	1	N/A	Bilateral	Partial D trisomy	X-ray	N/A	N/A	
	1	40	Male	1	Respiratoty distress	Bilateral	Tricuspid atresia, hypoplastic right ventricle	X-ray	Surgical	Death	
	1	40	Male	1	Diffuse hypotonia, mild cyanosis	Right	Facial diplegia, bilateral ptosis, diminished sucking reflex, myotonic dystrophy	X-ray	N/A	Multiple atelectasis and pneumonia	N/A
Leyten, 1992 [75]	1	37	Male	1	Upwards displacement of the right hemidiaphragm	Right	Facial dysmorphies	Intraoperative	Surgical	Good	No
Danhaive, 2007 [76]	1	40	Female	1	Respiratory failure	Right	No	A chest radiogram, laparotomy	Surgical	Death at 2 months of age	
Antoniou, 2012 [77]	1	37	Female	1	Respiratory failure	Left	Cystic mesenchymal hamartoma	Prenatal ultrasound, chest radiography, upper gastrointestinal contrast study	Surgical	N/A	Yes

Verloes, 2010 [78]	1	Term	Male	1	Neonatal respiratory distress	Left	Complete agenesis of the left diaphragm and absence of the left pericardium	Routine prenatal ultrasound scan	Surgical	Good	No
Inoue, 2020 [79]	1	39	Male	3	Asymptomatic	Left	No	Fetal ultrasonography, echocardiography, chest radiography, chest CT	Surgical at 8 months of life	Good	
	1	38	Male	1	Neonatal respiratory distress	Left	No	Fetal ultrasonography, prenatal MRI, chest radiograph, chest CT	Surgical at day 21	Good	No
Şahin, 2014 [80]	1	Term	Female	N/A	Neonatal respiratory distress	Right	Chromosomal anomalies	Fluoroscopy	N/A	Developmental retardation and intellectual disability at an age of 7 years old	No
Mahalingam, 2020 [81]	1	24	N/A	N/A	N/A	Left	N/A	Fetal MRI	Surgical	Death 1 month afer surgery	
	1	21	N/A	N/A	N/A	Left	N/A	Fetal MRI	Surgical	Doing well at 2 months follow-up	No
	1	32	N/A	N/A	N/A	Left	N/A	Fetal MRI	Conservative	Survived	No
	1	21	N/A	N/A	N/A	Left	N/A	Fetal MRI	Conservative	Survived	
Rodgers, 1986 [82]	1	39	Female	2	Severe respiratory insufficiency	Bilateral	None	Chest radiograph, chest fluoroscopy, and a radionuclide liver scan	Surgical	Good	No
	1	39	Female	1	Severe respiratory insufficiency	Bilateral	None	Chest radiograph, U/S	Surgical	Good	No
	1	29	Male	1	Severe respiratory insufficiency	Bilateral	None	Chest radiograph, U/S	Surgical	Recurrent episodes of pneumonia. At age 14 months, requires intermittent	No

										ventilatory support	
Zainudin, 2016 [83]	1	Term	Male	10	Significant respiratory insufficiency	Right	None referred	U/S, CT	Surgical	Good	No
Hasegawa, 1994 [84]	1	37	Female	1	Respiratory distress	Right	None	Fetal U/S, chest X-ray	Surgical	Good	No
N'guessen, 1984 [85]	1	Preterm	Male	N/A	N/A	Bilateral	Cloacal exstrophy, closed sacral meningocele and "butterfly" anomalies of T-5 to T-11 vertebrae	Chest X-ray, fluoroscopy	N/A	Death aged 23 days	
Takahashi, 2013 [86]	1	Term	Male	1	Respiratory distress	Right	None	Chest radiography and CT	Surgical	Good	No
Sun, 2016 [87]	1	38	Female	1	Respiratory insufficiency	Right	Asparagine synthetase deficiency (ASNSD)	Chest X-ray	Conservative	Death at 11 days of life	
Ahmed, 1998 [88]	1	39	Male	1	Respiratory insufficiency	Bilateral	None	Chest X-ray, U/S	Surgical	Good	No
	1	39	Female	1	Respiratory insufficiency, cyanosis	Bilateral	None	Chest X-ray	Surgical	Good	No
DiChiacchio, 2018 [89]	1	37	Male	1	Asymptomatic	Left	Extrapulmonary versus intrapulmonary sequestration with a systemic feeding vessel from the left internal mammary artery	Routine plain radiograph was obtained for confirmation of umbilical vein catheter placement, chest U/S	Conservative	Good	No

Park, 1992 [90]	1		Male	5	Vomiting, dyspnea	Left	Gastric volvulus	Abdominal radiographs and upper gastrointestinal series	Surgical		
Huang, 2021 [91]	1	36	Female	1	Respiratory insufficiency	Bilateral	Dextrocardia, left superior vena cava, omphaloceles	Abdominal radiographs	N/A	Death	
Veiga-Fernández, 2020 [92]	1	37	Female	Prenatal diagnosis	Marfanoid habitus	Right	Early-onset Marfan syndrome (EOMS)	Fetal U/S, fetal MRI	Conservative	Death after 3 months	
Jeanty, 2007 [93]	1	34	Female	Stillborn		Central	Pericardial effusion, multiple cardiac and skeletal anomalies	Prenatal U/S	Stillborn		

1. Singh, G.; Rai, R.K.; Pant, N.; Wakhlu, A. Study of Thoracoscopic Repair of Diaphragmatic Eventration in Children: A Case Series. *J Laparoendosc Adv Surg Tech A* **2020**, *30*, 692-694, doi:10.1089/lap.2019.0805.
2. Heiwegen, K.; van Heijst, A.F.; Daniels-Scharbatke, H.; van Peperstraten, M.C.; de Blaauw, I.; Botden, S.M. Congenital diaphragmatic eventration and hernia sac compared to CDH with true defects: a retrospective cohort study. *European journal of pediatrics* **2020**, *179*, 855-863, doi:10.1007/s00431-020-03576-w.
3. Hoshino, Y.; Arai, J. Diaphragm ultrasound examination for congenital diaphragmatic eventration in two premature neonates. *BMJ case reports* **2020**, *13*, doi:10.1136/bcr-2019-232813.
4. Iskender, C.; Tarım, E.; Yalcınkaya, C. Prenatal diagnosis of right diaphragmatic eventration associated with fetal hydrops. *The journal of obstetrics and gynaecology research* **2012**, *38*, 858-862, doi:10.1111/j.1447-0756.2011.01831.x.
5. Kadam, R.; Prasad, V. Intrathoracic Gastric Volvulus presenting with GIT Bleed. *Journal of neonatal surgery* **2017**, *6*, 40, doi:10.21699/jns.v6i2.489.
6. Kizilcan, F.; Tanyel, F.C.; Hiçsönmez, A.; Büyükpamukçu, N. The long-term results of diaphragmatic plication. *Journal of pediatric surgery* **1993**, *28*, 42-44, doi:10.1016/s0022-3468(05)80351-3.
7. Lai, C.Y.; Hsu, W.M.; Peng, S.S.; Chou, H.C.; Chen, C.Y.; Hsieh, W.S.; Tsao, P.N. Tubular jejunal duplication, amelia and congenital diaphragmatic eventration in a neonate. *Clinical dysmorphology* **2010**, *19*, 169-171, doi:10.1097/MCD.0b013e328339ab0c.
8. Gunadi; Balela, N.; Marcellus; Fauzi, A.R.; Dwihantoro, A. Eventration of right diaphragm with an intrathoracic ectopic kidney: A case report. *Annals of medicine and surgery (2012)* **2020**, *57*, 179-182, doi:10.1016/j.amsu.2020.07.034.
9. Kasdallah, N.; Ben Salem, H.; Kbaier, H.; Blibech, S.; Douagi, M. Congenital diaphragmatic eventration complicated with neonatal gastric perforation. *La Tunisie medicale* **2017**, *95*, 136-138.
10. Mitsiakos, G.; Gavras, C.; Katsaras, G.N.; Chatziioannidis, I.; Mouravas, V.; Mitsiakou, C.; Lampropoulos, V.; Nikolaidis, N. Parvovirus B19 Intrauterine Infection and Eventration of the Diaphragm. *Prague Med Rep* **2022**, *123*, 48-55, doi:10.14712/23362936.2022.6.
11. Revencu, N.; Quenum, G.; Detaille, T.; Verellen, G.; De Paepe, A.; Verellen-Dumoulin, C. Congenital diaphragmatic eventration and bilateral uretero-hydronephrosis in a patient with neonatal Marfan syndrome caused by a mutation in exon 25 of the FBN1 gene and review of the literature. *European journal of pediatrics* **2004**, *163*, 33-37, doi:10.1007/s00431-003-1330-8.
12. Sallout, B.; Alshebli, D.; Sallout, L.; Al Baqawi, B.; Faden, M.S. Fetal Diaphragmatic Eventration: A Case Report. *Journal of obstetrics and gynaecology Canada : JOGC = Journal d'obstetrique et gynecologie du Canada : JOGC* **2021**, *43*, 993-997, doi:10.1016/j.jogc.2020.12.014.
13. Tsukahara, Y.; Ohno, Y.; Itakura, A.; Mizutani, S. Prenatal diagnosis of congenital diaphragmatic eventration by magnetic resonance imaging. *American journal of perinatology* **2001**, *18*, 241-244, doi:10.1055/s-2001-16992.
14. Yang, J.I. Left diaphragmatic eventration diagnosed as congenital diaphragmatic hernia by prenatal sonography. *Journal of clinical ultrasound : JCU* **2003**, *31*, 214-217, doi:10.1002/jcu.10157.

15. Zankl, A.; Osterheld, M.C.; Vial, Y.; Beurret, N.; Meuli, R.; Meagher-Villemure, K.; Roth-Kleiner, M. Right-sided diaphragmatic eventration: a rare cause of non-immune hydrops fetalis. *Neonatology* **2007**, *92*, 14-18, doi:10.1159/000098412.
16. Lewis, L.; Poojari, G.; Sanoj, K.M.; Kamath, S.P.; Kachane, Y.P. Neonatal arrhythmia with diaphragmatic eventration. *Indian journal of pediatrics* **2008**, *75*, 1083-1085, doi:10.1007/s12098-008-0097-3.
17. Shukla, R.M.; Maitra, S.K.; Patra, M.P.; Mandal, K.C.; Sarkar, R.; Mukhopadhyay, B.; Bhattacharya, M. Eventration of diaphragm with gastric duplication cysts: a rare association. *Indian journal of pediatrics* **2012**, *79*, 1377-1379, doi:10.1007/s12098-012-0688-x.
18. Sathanandam, S.; Kumar, T.K.; Feliz, A.; Knott-Craig, C.J. Successful Repair of Hypoplastic Left Heart Syndrome With Intact Atrial Septum, Congenital Diaphragm Hernia, and Anomalous Origin of Coronary Artery: Defying the Odds. *The Annals of thoracic surgery* **2016**, *102*, e55-57, doi:10.1016/j.athoracsur.2015.12.052.
19. Shehata, S.M.; El-Banna, I.A.; Gaber, A.A.; El-Samongy, A.M. Spondylothoracic dysplasia with diaphragmatic defect: a case report with literature review. *European journal of pediatric surgery : official journal of Austrian Association of Pediatric Surgery ... [et al] = Zeitschrift fur Kinderchirurgie* **2000**, *10*, 337-339, doi:10.1055/s-2008-1072387.
20. Steiner, R.D.; St, J.D.P.; Hopkin, R.J.; Kozielski, R.; Bove, K.E. Combination of diaphragmatic eventration and microphthalmia/anophthalmia is probably nonrandom. *American journal of medical genetics* **2002**, *108*, 45-50, doi:10.1002/ajmg.10167.
21. Stratton, R.F.; Young, R.S.; Heiman, H.S.; Carter, J.M. Fryns syndrome. *American journal of medical genetics* **1993**, *45*, 562-564, doi:10.1002/ajmg.1320450507.
22. Symbas, P.N.; Hatcher, C.R., Jr.; Waldo, W. Diaphragmatic eventration in infancy and childhood. *The Annals of thoracic surgery* **1977**, *24*, 113-119, doi:10.1016/s0003-4975(10)63718-6.
23. Takahashi, K.; Miyake, A.; Nakayashiro, M. Hypoplastic left heart syndrome in PAGOD syndrome. *Pediatrics international : official journal of the Japan Pediatric Society* **2014**, *56*, 422-424, doi:10.1111/ped.12280.
24. Vieira Martins, M.; Martins, D.S.; Oliveira, G. Diaphragmatic eventration as a first sign of Scimitar syndrome. *Cardiol Young* **2021**, *31*, 1870-1872, doi:10.1017/s1047951121001694.
25. Vijayaraghavan, S.B. Diaphragmatic eventration into the pericardial sac: sonographic diagnosis. *J Clin Ultrasound* **1988**, *16*, 510-512, doi:10.1002/jcu.1870160711.
26. Wayne, E.R.; Burrington, J.D.; Myers, D.N.; Cotton, E.; Block, W. Letter: Bilateral eventration of the diaphragm in a neonate with congenital cytomegalic inclusion disease. *J Pediatr* **1973**, *83*, 164-165, doi:10.1016/s0022-3476(73)80339-7.
27. Byun, J.; Kim, H.Y.; Noh, S.Y.; Kim, S.H.; Jung, S.E.; Lee, S.C.; Park, K.W. Neonatal gastric perforation: A single center experience. *World J Gastrointest Surg* **2014**, *6*, 151-155, doi:10.4240/wjgs.v6.i8.151.
28. Snyder, C.W.; Walford, N.E.; Danielson, P.D.; Chandler, N.M. A simple thoracoscopic plication technique for diaphragmatic eventration in neonates and infants: technical details and initial results. *Pediatr Surg Int* **2014**, *30*, 1013-1016, doi:10.1007/s00383-014-3580-0.

29. Ratan, S.K.; Grover, S.B. Lung agenesis in a neonate presenting with contralateral mediastinal shift. *Am J Perinatol* **2001**, *18*, 441-446, doi:10.1055/s-2001-18788.
30. Rais-Bahrami, K.; Gilbert, J.C.; Hartman, G.E.; Chandra, R.S.; Short, B.L. Right diaphragmatic eventration simulating a congenital diaphragmatic hernia. *Am J Perinatol* **1996**, *13*, 241-243, doi:10.1055/s-2007-994372.
31. Sciorra, L.J.; Hux, C.; Day-Salvadore, D.; Lee, M.L.; Mandelbaum, D.E.; Brady-Yasbin, S.; Frybury, J.; Mahoney, M.J.; Dimaio, M.S. Trisomy 5 mosaicism detected prenatally with an affected liveborn. *Prenat Diagn* **1992**, *12*, 477-482, doi:10.1002/pd.1970120602.
32. Yang, C.K.; Shih, J.C.; Hsu, W.M.; Peng, S.S.; Shyu, M.K.; Lee, C.N.; Hsieh, F.J. Isolated right diaphragmatic eventration mimicking congenital heart disease in utero. *Prenat Diagn* **2005**, *25*, 872-875, doi:10.1002/pd.1281.
33. Poole, C.A.; Rowe, M.I.; Fojaco, R.M.; Wexler, H.A. Congenital eventration of the septum transversum: a rare form of congenital diaphragmatic anomaly. *Pediatr Radiol* **1976**, *4*, 257-260, doi:10.1007/bf02461536.
34. Kapoor, V.; Wright, I.M. Congenital myotonic dystrophy with cardiac conduction defect and eventration of the diaphragm. *Pediatr Int* **2010**, *52*, e6-8, doi:10.1111/j.1442-200X.2009.02998.x.
35. Pearl, M. Sequestration of the lung. *Am J Dis Child* **1972**, *124*, 706-707, doi:10.1001/archpedi.1972.02110170084013.
36. Renault, F.; Nicot, F.; Liptaï, Z.; Benharrats, T.; Fauroux, B. Congenital diaphragm weakness without neuromuscular disease. *Muscle Nerve* **2008**, *38*, 1201-1205, doi:10.1002/mus.20956.
37. Moerman, P.; Fryns, J.P.; Devlieger, H.; Van Assche, A.; Lauweryns, J. Congenital eventration of the diaphragm: an unusual cause of intractable neonatal respiratory distress with variable etiology. *Am J Med Genet* **1987**, *27*, 213-218, doi:10.1002/ajmg.1320270124.
38. Moccia, W.A.; Kaude, J.V.; Felman, A.H. Congenital eventration of the diaphragm. Diagnosis by ultrasound. *Pediatr Radiol* **1981**, *10*, 197-200, doi:10.1007/bf01001581.
39. Moazam, F.; Rodgers, B.M.; Talbert, J.L. Use of Teflon mesh for repair of abdominal wall defects in neonates. *J Pediatr Surg* **1979**, *14*, 347-351, doi:10.1016/s0022-3468(79)80497-2.
40. Gupta, S.; Wyllie, J.; Wright, C.; Turnbull, D.M.; Taylor, R.W. Mitochondrial respiratory chain defects and developmental diaphragmatic dysfunction in the neonatal period. *J Matern Fetal Neonatal Med* **2006**, *19*, 587-589, doi:10.1080/14767050600632623.
41. Merten, D.F.; Bowie, J.D.; Kirks, D.R.; Grossman, H. Anteromedial diaphragmatic defects in infancy: current approaches to diagnostic imaging. *Radiology* **1982**, *142*, 361-365, doi:10.1148/radiology.142.2.7054823.
42. Meine, F.; Grossman, H.; Forman, W.; Jackson, D. The radiographic findings in congenital cutis laxa. *Radiology* **1974**, *113*, 687-690, doi:10.1148/113.3.687.
43. Macayran, J.F.; Doroshow, R.W.; Phillips, J.; Sinow, R.M.; Furst, B.A.; Smith, L.M.; Lin, H.J. PAGOD syndrome: eighth case and comparison to animal models of congenital vitamin A deficiency. *Am J Med Genet* **2002**, *108*, 229-234, doi:10.1002/ajmg.10262.

44. Kozlov, Y.; Novozhilov, V. Thoracoscopic plication of the diaphragm in infants in the first 3 months of life. *J Laparoendosc Adv Surg Tech A* **2015**, *25*, 342-347, doi:10.1089/lap.2014.0205.
45. Joshi, A.; Kumar, M.; Acharya, A. Bilateral congenital eventration of diaphragm: keep in mind, the other side. *BMJ Case Rep* **2018**, *2018*, doi:10.1136/bcr-2018-226051.
46. N, S.; R, R.; H, S. Thoracoscopic Plication for Bilateral Eventration of Diaphragm in a Neonate. *J Neonatal Surg* **2015**, *4*, 35.
47. Heo, J.S.; Song, J.Y.; Choi, E.Y.; Kim, E.H.; Kim, J.H.; Park, S.E.; Jeon, J.H. Atypical Neonatal Marfan Syndrome with p.Glu1073Lys Mutation of FBN1: the First Case in Korea. *J Korean Med Sci* **2017**, *32*, 1-3, doi:10.3346/jkms.2017.32.1.1.
48. Gerard-Blanluet, M.; Port-Lis, M.; Baumann, C.; Perrin-Sabourin, L.; Ebrad, P.; Audry, G.; Delezoide, A.L.; Verloes, A. Unilateral agenesis of the abdominal wall musculature: An early muscle deficiency. *Am J Med Genet A* **2010**, *152a*, 2870-2874, doi:10.1002/ajmg.a.33708.
49. Garbaccio, C.; Gyepes, M.T.; Fonkalsrud, E.W. Malfunction of the intact diaphragm in infants and children. *Arch Surg* **1972**, *105*, 57-61, doi:10.1001/archsurg.1972.04180070055011.
50. Falade, A.G. Right diaphragmatic eventration simulating neonatal pleural effusion: a case report. *Ann Trop Paediatr* **1992**, *12*, 221-223, doi:10.1080/02724936.1992.11747572.
51. Ertan, G.; Sinzig, M.; Tekes, A.; Huisman, T.A. Congenital intercostal liver herniation: report on two neonates. *Eur J Pediatr Surg* **2010**, *20*, 196-198, doi:10.1055/s-0029-1237733.
52. Elberg, J.J.; Brok, K.E.; Pedersen, S.A.; Kock, K.E. Congenital bilateral eventration of the diaphragm in a pair of male twins. *J Pediatr Surg* **1989**, *24*, 1140-1141, doi:10.1016/s0022-3468(89)80096-x.
53. Ekkelkamp, S.; Vos, A. Bilateral eventration of the diaphragm. *Z Kinderchir* **1983**, *38*, 412-413, doi:10.1055/s-2008-1060020.
54. Rana, A.; Manchanda, S.; Dadhwal, V. Congenital focal eventration of the left hemidiaphragm: diagnostic dilemma resolved on prenatal MRI. *BMJ Case Rep* **2021**, *14*, doi:10.1136/bcr-2021-245771.
55. Dalvi, R.; Chari, G.; Fernandez, A.R. Congenital eventration of diaphragm. *Indian J Pediatr* **1990**, *57*, 125-127, doi:10.1007/bf02722146.
56. Krasemann, T.; van Osch-Gevers, L.; van de Woestijne, P. Cyanosis due to an isolated atrial septal defect: case report and review of the literature. *Cardiol Young* **2020**, *30*, 1741-1743, doi:10.1017/s1047951120002656.
57. Comstock, C.H. The antenatal diagnosis of diaphragmatic anomalies. *J Ultrasound Med* **1986**, *5*, 391-396, doi:10.7863/jum.1986.5.7.391.
58. Wenger, S.L.; McPherson, E.W. Interstitial deletion 2(p11.2p13): a rare chromosomal abnormality. *Clin Genet* **1997**, *52*, 61-62, doi:10.1111/j.1399-0004.1997.tb02516.x.
59. Donahue, M.L.; Rohena, L.O. Rare presentation of 6q16.3 microdeletion syndrome with severe upper limb reduction defects and duodenal atresia. *Clin Case Rep* **2017**, *5*, 905-914, doi:10.1002/ccr3.916.
60. Othersen, H.B., Jr.; Lorenzo, R.L. Diaphragmatic paralysis and eventration: newer approaches to diagnosis and operative correction. *J Pediatr Surg* **1977**, *12*, 309-315, doi:10.1016/0022-3468(77)90006-9.

61. Ein, S.H. Imperforate anus (anal agenesis) with rectal and sigmoid atresias in a newborn. *Pediatr Surg Int* **1997**, *12*, 449-451, doi:10.1007/bf01076966.
62. Berdon, W.E.; Baker, D.H.; Amoury, R. The role of pulmonary hypoplasia in the prognosis of newborn infants with diaphragmatic hernia and eventration. *Am J Roentgenol Radium Ther Nucl Med* **1968**, *103*, 413-421, doi:10.2214/ajr.103.2.413.
63. Shahrul Baharin, N.; Awadh Hashim, E.; Bin Huey, Q.; Chandran, S. Reinforcing the vascular disruption theory of the genesis of Poland's syndrome: a rare association of diaphragmatic eventration in a preterm infant with severe musculoskeletal defects. *BMJ Case Rep* **2021**, *14*, doi:10.1136/bcr-2020-238392.
64. Buxi, T.B.; Shubhra; Khanna, V.K.; Saxena, U.; Dutta, T. CT in complete congenital eventration of diaphragm with aplasia of lung. *Australas Radiol* **1992**, *36*, 72-74, doi:10.1111/j.1440-1673.1992.tb03082.x.
65. Iliff, P.J.; Eyre, J.A.; Westaby, S.; de Leval, M.; de Sousa, C. Neonatal pericardial effusion associated with central eventration of the diaphragm. *Arch Dis Child* **1983**, *58*, 147-149, doi:10.1136/adc.58.2.147.
66. Rao, S.H.; Natarajan, S.; Pallavi, V. Anesthetic management of staged thoracoscopic repair of bilateral eventration of diaphragm in a neonate. *Anesth Essays Res* **2016**, *10*, 118-121, doi:10.4103/0259-1162.165497.
67. Andijani, A.A.; Shajira, E.S.; Abushaheen, A.; Al-Matary, A. Microphthalmia Syndrome 9: Case Report of a Newborn Baby with Pulmonary Hypoplasia, Diaphragmatic Eventration, Microphthalmia, Cardiac Defect and Severe Primary Pulmonary Hypertension. *Am J Case Rep* **2019**, *20*, 354-360, doi:10.12659/ajcr.912873.
68. Baker, E.K.; Al Gharaibeh, F.N.; Bove, K.; Calvo-Garcia, M.A.; Shillington, A.; VandenHeuvel, K.; Cortezzo, D.E. A novel RYR1 variant in an infant with a unique fetal presentation of central core disease. *Am J Med Genet A* **2023**, doi:10.1002/ajmg.a.63188.
69. Chitayat, D.; Sroka, H.; Keating, S.; Colby, R.S.; Ryan, G.; Toi, A.; Blaser, S.; Viero, S.; Devisme, L.; Boute-Bénéjean, O.; et al. The PDAC syndrome (pulmonary hypoplasia/agenesis, diaphragmatic hernia/eventration, anophthalmia/microphthalmia, and cardiac defect) (Spear syndrome, Matthew-Wood syndrome): report of eight cases including a living child and further evidence for autosomal recessive inheritance. *Am J Med Genet A* **2007**, *143a*, 1268-1281, doi:10.1002/ajmg.a.31788.
70. Kosho, T.; Nakamura, T.; Kawame, H.; Baba, A.; Tamura, M.; Fukushima, Y. Neonatal management of trisomy 18: clinical details of 24 patients receiving intensive treatment. *Am J Med Genet A* **2006**, *140*, 937-944, doi:10.1002/ajmg.a.31175.
71. Robinson, A.E.; Gooneratne, N.S.; Blackburn, W.R.; Brogdon, B.G. Bilateral anteromedial defect of the diaphragm in children. *AJR Am J Roentgenol* **1980**, *135*, 301-306, doi:10.2214/ajr.135.2.301.
72. Miyano, G.; Morita, K.; Kaneshiro, M.; Miyake, H.; Koyama, M.; Nouso, H.; Yamoto, M.; Nakano, R.; Tanaka, Y.; Nishiguchi, T.; et al. Unilateral pulmonary agenesis associated with oesophageal atresia and tracheoesophageal fistula: A case report with prenatal diagnosis. *Afr J Paediatr Surg* **2015**, *12*, 86-88, doi:10.4103/0189-6725.151000.

73. Arts, P.; Garland, J.; Byrne, A.B.; Hardy, T.S.E.; Babic, M.; Feng, J.; Wang, P.; Ha, T.; King-Smith, S.L.; Schreiber, A.W.; et al. Paternal mosaicism for a novel PBX1 mutation associated with recurrent perinatal death: Phenotypic expansion of the PBX1-related syndrome. *Am J Med Genet A* **2020**, *182*, 1273-1277, doi:10.1002/ajmg.a.61541.
74. Wexler, H.A.; Poole, C.A. Neonatal diaphragmatic dysfunction. *AJR Am J Roentgenol* **1976**, *127*, 617-622, doi:10.2214/ajr.127.4.617.
75. Leyten, Q.H.; Renier, W.O.; Gabreëls, F.J.; ter Laak, H.J.; Hinkofer, L.H. Dystrophic myopathy of the diaphragm in a neonate with severe respiratory failure during infectious episodes. *Neuromuscul Disord* **1993**, *3*, 51-55, doi:10.1016/0960-8966(93)90041-h.
76. Danhaive, O.; Lozzi, S.; D'Amico, A.; Devito, R.; Boldrini, R.; Corchia, C.; Bagolan, P.; Bertini, E. Neonatal-onset nemaline myopathy mimicking congenital diaphragmatic hernia. *J Pediatr Surg* **2007**, *42*, E19-22, doi:10.1016/j.jpedsurg.2007.04.030.
77. Antoniou, D.; Soutis, M.; Karentzou, O. Cystic mesenchymal hamartoma arising in intrathoracic heterotopic liver: a case report. *J Pediatr Surg* **2012**, *47*, e49-51, doi:10.1016/j.jpedsurg.2012.07.057.
78. Verloes, A.; Perrin, L.; Delbecque, K.; Gonzales, M.; Demarche, M.; Dekoster, G. Congenital absence of the left pericardium and diaphragmatic defect in sibs. *Eur J Med Genet* **2010**, *53*, 133-135, doi:10.1016/j.ejmg.2010.02.007.
79. Inoue, S.; Odaka, A.; Muta, Y.; Takeuchi, Y.; Yamashita, T.; Kabe, K.; Sakurai, Y. Left hemidiaphragmatic elevation: Value of histology. *Journal of Pediatric Surgery Case Reports* **2020**, *60*, doi:10.1016/j.epsc.2020.101546.
80. Şahin, Y.; Kiper, P.; Alanay, Y.; Liehr, T.; Utine, G.E.; Boduroğlu, K. Partial monosomy 3q26.33-3q27.3 presenting with intellectual disability, facial dysmorphism, and diaphragm eventration: a case report. *Clin Dysmorphol* **2014**, *23*, 147-151, doi:10.1097/mcd.0000000000000042.
81. Mahalingam, H.; Babu, B.; Rangasami, R.; Suresh, S.; Suresh, I.; Andrew, C. Fetal magnetic resonance imaging in the evaluation of congenital diaphragmatic anomalies. *Egyptian Journal of Radiology and Nuclear Medicine* **2020**, *51*, doi:10.1186/s43055-020-00340-w.
82. Rodgers, B.M.; Hawks, P. Bilateral congenital eventration of the diaphragms: successful surgical management. *J Pediatr Surg* **1986**, *21*, 858-864, doi:10.1016/s0022-3468(86)80008-2.
83. Zainudin, Z.; Abdullah, F.I.S.; Hong, N.S.; Bahari, M.; Sim, I.C.G. A "near miss" congenital eventration of the right hemidiaphragm in a neonate: A case report. *Malaysian Journal of Medicine and Health Sciences* **2016**, *12*, 45-48.
84. Hasegawa, T.; Imura, K.; Kubota, A.; Kobayashi, T.; Inoue, M.; Hata, S.; Shimizu, I.; Suehara, N. Congenital diaphragmatic eventration detected by antenatal ultrasound: Rationale for early operation. *Pediatric Surgery International* **1994**, *9*, 405-406, doi:10.1007/BF01686016.
85. N'Guessen, G.; Stephens, F.D.; Pick, J. Congenital superior ectopic (thoracic) kidney. *Urology* **1984**, *24*, 219-228, doi:10.1016/0090-4295(84)90346-7.
86. Takahashi, T.; Okazaki, T.; Ochi, T.; Nishimura, K.; Lane, G.J.; Inada, E.; Yamataka, A. Thoracoscopic plication for diaphragmatic eventration in a neonate. *Ann Thorac Cardiovasc Surg* **2013**, *19*, 243-246, doi:10.5761/atcs.cr.12.01907.
87. Sun, J.; McGillivray, A.J.; Pinner, J.; Yan, Z.; Liu, F.; Bratkovic, D.; Thompson, E.; Wei, X.; Jiang, H.; Asan; et al. Diaphragmatic Eventration in Sisters with Asparagine Synthetase Deficiency: A Novel Homozygous ASNS Mutation and Expanded Phenotype. *JIMD Rep* **2017**, *34*, 1-9, doi:10.1007/8904_2016_3.

88. Ahmed, Z.; Mohyuddin, Z. Congenital bilateral diaphragmatic eventration--a rare anomaly with complicated perioperative care. *J Pak Med Assoc* **1998**, *48*, 221-225.
89. DiChiacchio, L.; Cappiello, C.D.; Greenspon, J. Extrapulmonary sequestration with a left internal thoracic arterial feeding vessel in an infant treated with video-assisted Thoracoscopic resection: a case report. *J Cardiothorac Surg* **2018**, *13*, 88, doi:10.1186/s13019-018-0775-9.
90. Park, W.H.; Choi, S.O.; Suh, S.J. Pediatric gastric volvulus--experience with 7 cases. *J Korean Med Sci* **1992**, *7*, 258-263, doi:10.3346/jkms.1992.7.3.258.
91. Huang, X.; Huang, H.; Liang, Y.; Jiang, H.; Pei, Y.; Xu, L.; Tang, W.; Li, Y. Modified sequential sac ligation and staged closure technique for the management of giant omphalocele. *J Pediatr Surg* **2021**, *56*, 1576-1582, doi:10.1016/j.jpedsurg.2020.11.031.
92. Veiga-Fernández, A.; Joigneau Prieto, L.; Álvarez, T.; Ruiz, Y.; Pérez, R.; Gámez, F.; Ortega Abad, V.; Yllana, F.; De León-Luis, J. Perinatal diagnosis and management of early-onset Marfan syndrome: case report and systematic review. *J Matern Fetal Neonatal Med* **2020**, *33*, 2493-2504, doi:10.1080/14767058.2018.1552935.
93. Jeanty, C.; Nien, J.K.; Espinoza, J.; Kusanovic, J.P.; Gonçalves, L.F.; Qureshi, F.; Jacques, S.; Lee, W.; Romero, R. Pleural and pericardial effusion: a potential ultrasonographic marker for the prenatal differential diagnosis between congenital diaphragmatic eventration and congenital diaphragmatic hernia. *Ultrasound Obstet Gynecol* **2007**, *29*, 378-387, doi:10.1002/uog.3958.
